# Medical grade honey: Hope for wounded white rhinos

**DOI:** 10.1016/j.vas.2021.100196

**Published:** 2021-08-18

**Authors:** Hendrik J. Marais, Zoe G. Glyphis, Niels A.J. Cremers

**Affiliations:** a Saving the Survivors, Pretoria, South Africa; b Department of Companion Animal Clinical Studies, Faculty of Veterinary Science, University of Pretoria, 0110 Onderstepoort, South Africa; c Triticum Exploitatie BV, Sleperweg 44, 6222NK Maastricht, the Netherlands

**Keywords:** Rhinos, Wildlife management, Conservation medicine, Medical grade honey, Wounds, Wound Healing

## Abstract

South Africa is home to some of the world's most endangered wildlife, with the White rhinoceros (*Cerathotherium simum*) among its most threatened species due to its highly sought-after horn. Since the reproduction number is below the mortality rate, there is a decrease in rhinos year over year, urgently requiring changes and saving the survivors. In this study, the efficacy of medical grade honey (MGH) for wound care in rhinos was investigated.

We presented a case series of seven rhinos with wounds of different etiologies, including gunshots and poaching. Four wounds were around the horns and three on the limbs. It was a challenge to take care and follow-up the wounds of these wild animals in their natural habitat. A well-balanced decision between the need of treatment and risk of immobilization is constantly evaluated. In the presented cases, MGH proved a novel tool with minimal intervention and maximal effectiveness. With the severity of the wounds taken into consideration, there was a rapid healing in all cases, while infection resolved when present. MGH creates a moist and anti-inflammatory wound environment, while promoting almost all aspects in the wound healing processes, such as autolytic debridement, angiogenesis and re-epithelialization. The efficacy of MGH has constantly been confirmed in other cases and literature as well. MGH forms a potent therapy for treating wounded rhinos, independent of the severity of the wound. The strong antimicrobial and healing properties make it an easy and versatile product that can be used in all kinds of wounds.

## Highlights

• Rhinos are endangered by poachers, often resulting in severe wounds or death.
• Limited data on wound management in wildlife exists.
• Medical grade honey exerts strong antimicrobial and healing properties.
• Medical grade honey can improve wound healing and survival of rhinos.
• The manuscript covers extensive literature data to support the clinical findings.

## 1 Introduction

The third largest herbivore and largest species of the Rhinocerotidae is the White rhinoceros (*Ceratotherium simum*) (Plochocki, Ruiz, Rodriguez-Sosa & Hall, 2017). This megaherbivore was not always a victim of human persecution, such that after becoming extinct in 1896 at Kruger National Park and finally being reintroduced in the 1960′s, its numbers increased to almost 20.000 steadily until 2008 (Ferreira et al., 2015). From then, poaching in South Africa has increased dramatically from 83 to 1215 cases in 2014 due to a high demand for rhino horn and subsequently the population of rhinos declined accordingly (Ferreira, Botha & Emmett, 2012; Haas & Ferreira, 2016; "https://www.savetherhino.org/rhino-info/poaching-stats/ (accessed on September 29, 2020),"; Ververs et al., 2017). This number has gradually decreased since then to 1028, 769 and 594 in 2017, 2018, and 2019, respectively ("https://www.savetherhino.org/rhino-info/poaching-stats/ (accessed on September 29, 2020),"). It seems like raising awareness of this problem, preventive removal of the horns, international ban on horn trading and trafficking, fighting corruption, strict government measures, and efforts in arresting poachers are taking effect. However, because the poaching and natural mortality rates due to drought outpaced the birth of new rhinos, less rhinos are alive and this makes it harder for poachers to locate their prey (Ferreira, le Roex & Greaver, 2019). On average, rhinos give first birth when they are about 7-years of age and with a pregnancy time ranging between 16 and 18 months and inter-calving time of 2.5 year (Ververs et al., 2017). The population of White rhinos in the Kruger National Park was estimated to be 5142 (95% CI: 4759–5532) in September 2017 (Ferreira et al., 2019). To illustrate, in the one-year study period before September 2017, the number of poached White rhinos in Kruger National Park was 513, the mortality by natural cause was 118 and unknown cause 20, while 13 rhinos died by lethal removal and 13 were non-lethally removed from the park (Ferreira et al., 2019). In sharp contrast to the total decrease of 678 rhinos, only 345 were estimated to be born, resulting in a net decrease of 333 rhinos (Ferreira et al., 2019).

Since rhinos are left for dead after the poaching incidents, it is of utmost importance to save the survivors. The reason that poachers do not kill the animals directly is usually because of poor shot placement and poachers being inexperienced and they do not want to alert the rangers and people on the reserve by firing an extra shot. When using veterinary drugs for sedation, poachers use it illegally and they are not trained in using it or the reversal drug while often having limited access. The wounds that arise following poaching differ in severity and location, depending on the poachers using guns or dart tranquilizers, and the different methods of removal the horns, with either chainsaws, machetes, hatchets, or saws. Conservationists and veterinarians are among those on the frontline dealing with these injured animals on a day-to-day basis, and therefore it is important to optimize treatment options and improve wound care. Since an evidence-based wound care protocol is lacking for treating these majestic animals, more information on potent wound care protocols is urgently warranted.

There are several challenges to be overcome by wildlife veterinarians for properly treating these wounds. It will always be tried to leave the animals in their natural habitat whenever possible and when the severity of the wound allows it. Rhinos do not always adapt well to living in a boma and this stress can result in the animal not eating, demanding a direct release despite their injuries. Since tracking of the animals can be time-consuming and both animal and environmental conditions sometimes do not allow immobilization, it is hard to provide the animals with regular wound care and continuously monitor the healing trajectory. In addition, clinical parameters cannot always be assessed in the best possible ways in the wild. A wound care product that allows irregular treatment intervals and having a long-term efficacy would help to overcome these challenges.

Honey has been used for wound healing since ancient times because of its antimicrobial and healing activities, and the first descriptions date back to 2600–2200 BC in Egypt (Eteraf-Oskouei & Najafi, 2013; Smaropoulos & Cremers, 2021). After antibiotics replaced the use of honey, the rise in antibiotic resistance has re-introduced medical grade honey (MGH) (Hermanns et al., 2020; Molan, 2002). MGH follows strict criteria to guarantee safety, quality, and efficacy, e.g. honey used for medical purposes must be free of contaminants and toxic substances, such as herbicides, pesticides, heavy metals, and microorganisms (Hermanns et al., 2020). In addition, to collecting the honey under organic conditions the honey needs to be sterilized using gamma irradiation to kill endospores and other microorganisms (Hermanns et al., 2020; Postmes, van den Bogaard & Hazen, 1995). Honey purchased at a beekeeper is not extensively tested and may be contaminated, while honey at the supermarket is often sterilized with heat, inactivating the enzymes contributing to the medicinal activities (Hermanns et al., 2020). The role of MGH in wound healing is binary and based on the antimicrobial and healing properties.

In contrast to modern antibiotics, which target specific cell mechanisms or structures, MGH holds broad-spectrum activity attributed to the more than 200 different constituents and its intrinsic properties. Since the antimicrobial activity rests on multiple mechanisms, there is no chance for the microorganisms to develop resistance towards MGH (Maddocks & Jenkins, 2013). MGH has a low pH that impedes pathogens to thrive, the hygroscopicity leads to dehydration of the microorganisms, and the constant release of low amounts of hydrogen peroxide, following catabolism of glucose by glucose oxidase, which is a potent antimicrobial. In addition, honey contains several substances that have a direct antioxidative and antimicrobial activity, such as the bee product propolis, bee-defensin-1 (antibacterial peptide secreted from bees to honey), and different phytochemicals, including chrysin, methylglyoxal, pinocembrin, pinobanksin, galangin, quercetin, lutelin, and kaemferol (Eteraf-Oskouei & Najafi, 2013; Hermanns, Cremers, Leeming & van der Werf, 2019; Mohapatra, Thakur & Brar, 2011; Nair, Tatavilis, Pospisilova, Kucerova & Cremers, 2020).

The healing properties of MGH are also based on multiple mechanisms. MGH creates a moist and more regenerative wound environment, has anti-oxidative and anti-inflammatory activity, and promotes autolytic debridement, angiogenesis and reepithelialization (E. Smaropoulos & N. A. Cremers, 2020; E. Smaropoulos & N. A. J. Cremers, 2020; Nair et al., 2020; Smaropoulos & Cremers, 2019, 2021).

Combining the aforementioned factors of rising poaching injuries with the challenges of treating patients in their natural habitat we explore new strategies to obtain the best possible results in the face of adversities. Unfortunately, not much data exists on wound care in rhinos, due to the difficulties to treat them without immobilization and to follow-up. Since these are wild animals, it always is a balance between necessity of care and the risk of immobilization. A simple wound care product that holds long-lasting antimicrobial and healing activity is therefore warranted. In this study, the efficacy of MGH for wound care in rhinos was supported by a prospective case series of seven wounds of different etiologies and severity.

## 2 Methods

### 2.1 Animals

“Saving the Survivors” is a non-profit organization that consists of a team of wildlife veterinarians whose mission is to save every animal who has fallen victim to poaching or traumatic incident. For example, Saving the Survivors takes care of White rhinos (*Ceratotherium simum*) that are injured and in need of treatment.

A case series of seven rhinos with injuries of different etiologies and severity was collected prospectively. The injuries ranged from gunshot wounds, pressure ulcers, to severe poaching wounds, and wounds were present around the legs (*n* = 3) or around the place of the horn (*n* = 4). An overview of the presented cases is given in Table 1.

**Table 1:** Cases overview.

Case number	Age and gender	Wound location	Cause of the wound
1	Adult cow	Foot	Unknown
2	Subadult cow	Limb	Gunshot
3	Adult bull	Leg	Pressure ulcer by cast for the treatment of fractures of metacarpus
4	Adult cow	Base of horn	Unknown origin
5	18-month Heifer	Broken horn	Unknown
6	2-year-old bull	Horns removed	Gunshot+ hatchet
7	5-year-old cow	Horns removed	Gunshot and possibly chainsaw or panga (machete)

Best possible care was given under circumstances in the wild. For this study, we selected seven rhinos that had typical wounds of different etiology and severity and were treated with MGH to demonstrate the efficacy and simplicity of this treatment. l-Mesitran Soft (Triticum Exploitatie BV, Maastricht, the Netherlands) was provided free of charge to Saving the Survivors and used for the wound care of these rhinos. Treatments were performed when possible and when time allowed. Since the rhinos are often still in the wild, follow up treatments and monitoring may be difficult and treatments intervals will be irregular. In addition, the necessity of a close follow-up of the healing and wound care treatment will be balanced to the condition of the animal and the risk of anesthesia and immobilization.

### 2.2 Anesthesia

Rhino anesthesia is carried out using a dartgun that fires a dart containing a mixture of a potent opioid (M99 – Etorphine hydrochloride) and a sedative such as azaperone or midazolam. Once the rhino is recumbent it is often partially reversed using butorphanol – an opioid agonist-antagonist, this lightens the anesthetic plane and reduces opioid-induced respiratory depression in rhino.

Because rhino are large animals and they suffer hypoxic muscle damage if they lie on their legs for too long, they need to constantly be ‘tipped’ from side to side under anesthesia or alternatively they need to lie on their side. White rhinos are notoriously sensitive to opioid drugs and immobilizations lasting longer than 45 min to an hour are not ideal but sometimes necessary to treat extensive wounds. Some rhinos, like “Hope” (case 7) were kept under anesthesia for up to 3 h for a treatment with very careful monitoring and constant weight shifting off of her limbs to ensure adequate circulation. Once a procedure is finished the rhino is given naltrexone which reverses the opioid, and the rhino is back on its feet within approximately 5 min or less.

A rhino will never be anesthetized if there is any indication that the risk outweighs the benefit. This includes immobilization when environmental conditions are not ideal i.e., too hot, too wet where the rhino can slip and injure itself or struggle to stand after anesthesia. If the terrain is not ideal and there is a possibility the rhino can injure itself, e.g., near water in which it can drown, the animal must move to a better area first. If there are other rhinos in the area that can injure the sedated rhino i.e., two bulls then this must be considered first. A cow with a calf on a reserve with predators such as lions, care must be taken to protect the calf if the mother is being treated, or the sedated mother is unable to care for the calf until the drug side effects wear off.

### 2.3 Radiographs

Radiographs were taken using a portable DR. system (Radmedix Acuity CSI). This system is completely portable, and the radiographs are immediately available on a laptop and allows to make treatment decision directly in the field. Radiographs were taken for suspected fractures and to sometimes look for bullets. They were also taken to assess damage and monitor progress of facial wounds.

### 2.4 Wound care treatment

Standard wound treatment protocol was to thoroughly clean and debride the wound using water, and chlorhexidine soap. All necrotic tissue would be debrided away followed by MGH (L-Mesitran Soft, Theo Manufacturing B.V., Maastricht, the Netherlands) application. l-Mesitran Soft was spread as a quite thick layer (2–4 mm on an even surface, and even thicker, up to 1 cm, in a pocket) to ensure a sustainable, long-lasting presence and activity. Zinc-Calcium alginate dressing was placed on top when it was heavily bleeding and otherwise regular calcium alginate (Covidien Kendall). The wound was subsequently covered with whatever the most suitable ‘bandage’ was to keep the dressings in place and to prevent contamination with dirt. This varied from casting material, to pieces of canvas and leather. The dressings were usually replaced every 4 to 6 weeks but they usually were off within 7 to 10 days due to the rhino rubbing them off. Dental acrylic was used for wounds at the base of horns as it is moldable and when it dries it is extremely hard and can be kept in place by drilling a hole in it and using wires for fixation.

The huge face wounds were initially covered in layers of soft casting material (BSN soft cast) which hardens when drying and this allowed the dressing to be molded to the wound. The dressing was then secured via two methods. In areas where there was still underlying bone, surgical screws were drilled through the fiberglass and into the bone to anchor it. In the areas with no underlying bone the dressing was secured using stainless steel wire as a suture material to suture the dressing to the skin. The same method was later used to secure pieces of leather over the dressing. This was a cheaper method and was quicker and seem to be tolerated better by the rhino then the rigid fiberglass. Unfortunately, none of these dressings lasted very long as these rhinos weigh upwards of 1,5 tons and they have huge force behind them to rub these dressings off if they irritate them.

Fractures of the legs or toe (metacarpal and metatarsal) were casted. Casts were well tolerated provided they were placed correctly and did not impede movement too much. Bullet holes were swabbed and cultured and most of the cultures came back showing that the bacteria present were resistant to most antibiotics.

Rhinos that were not clinically ill were never routinely given antibiotics. When needed Excede (Ceftiofur, Zoetis), Excenel (ceftiofur, Zoetis), Baytril (Enrofloxacin, Bayer) and Draxxin (tulathromycin, Zoetis) were used but normally based on the result of culture and antibiogram were possible.

### 2.5 Case reports

**Fig. 1:**
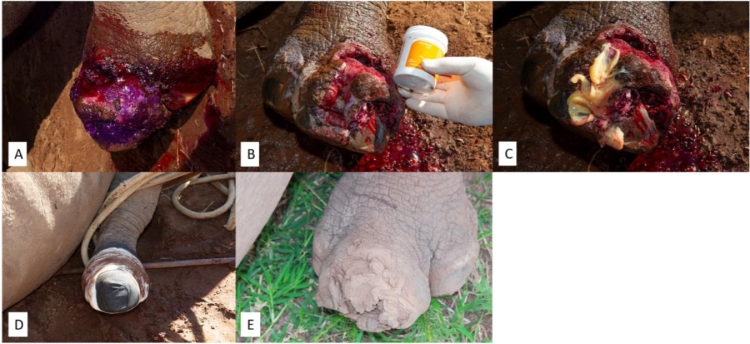
**Wound on the left hind foot. A).** Wound upon first presentation and cleaned with water and the antiseptic gentian violet. **B).** The wound was treated with MGH. **C).** MGH on the wound. **D).** After MGH application, the wound was bandaged to make sure the MGH stayed in place and no dirt could come in. **E).** Complete healing of the wounds after three treatments with MGH in three months’ time.

**Case 1:** An adult White Rhinoceros cow presented with a wound on her left hind foot (Fig. 1A). The origin of the wound was not certain. The rhino was immobilized, and the wound was cleaned with water and subsequently gentian violet as an antiseptic. Next, the wound was treated with MGH (Fig. 1B and C) and the wound was bandaged (Fig. 1D) before the rhino was released back in the wild. MGH was applied after 2 weeks and again after another 4 weeks. After only three MGH applications in three months’ time, the wound has completely healed (Fig. 1E).

**Fig. 2:**
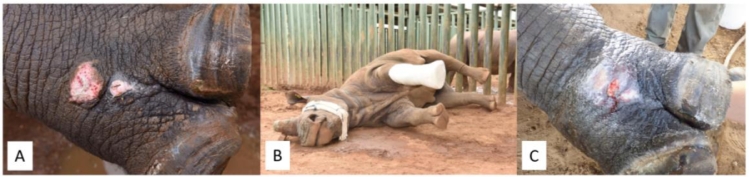
**An infected gunshot wound on the left anterior lower limb. A).** Photo of the wound at the start of the treatment with MGH in combination with alginate. **B).** The leg was casted after the treatment of the wound. **C).** Clear progression of the wound healing, 5 days after starting the treatment.

**Case 2:** A sub-adult White Rhinoceros cow presented with a 2-week-old infected wound resulting from a gunshot to the left anterior lower limb (Fig. 2A). Upon presentation, a swab was taken to determine the cause of the infection and antibiogram. The wounds were flushed with saline and the MGH was applied directly to the wounds and covered with alginate dressing and the limb was subsequently enclosed in a cast (Fig. 2B). The aggressive nature of the cow led to cracking of the cast within five days. Prior to fitting a thicker cast, visualization of the wounds revealed remarkable healing. At this time, the results of the swab were just returned, showing *Enterococcus faecalis* and *Aeromonas hydrophile*, and sensitivity towards Florfenicol, Ceftiofur and Enrofloxacin (Fig. 2C). Since the antimicrobial activity of the MGH resolved the infection and the wound healed so well by the MGH, the use of antibiotics was not needed anymore and thus prevented. The wound completely healed within the next 2 weeks, according to the referring veterinarian.

**Fig. 3:**
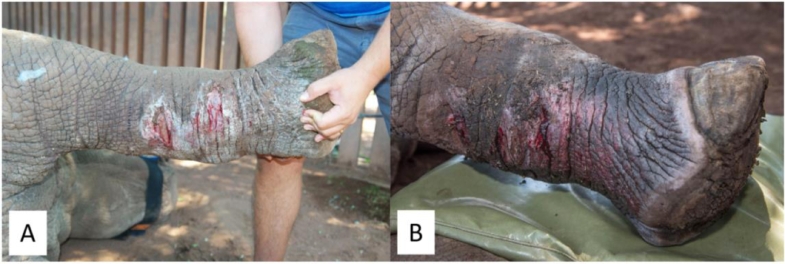
Pressure sore on the right anterior leg following the wearing of a cast for the treatment of metacarpus fractures. A). Pressure sores located on the lower limb at the start of MGH treatment. B). Wound progression and decrease in inflammation after 5 days of treatment with MGH.

**Case 3:** An adult White Rhinoceros bull presented with severe fractures of metacarpus 2 and 3 in the right anterior limb. To bull received a cast for treating the fractures. After four weeks, the cast was removed, and it was revealed that the cast caused some pressure ulcers on the leg (Fig. 3A). The pressure ulcers were treated with MGH and after five days, the wound has healed remarkably and inflammation was strongly reduced, particularly noticeable at the wound margins (Fig. 3B). Without the cast, the bull avoided weight bearing on the limb, and a new cast had to be placed to facilitate further healing of the fractures.

**Fig. 4:**
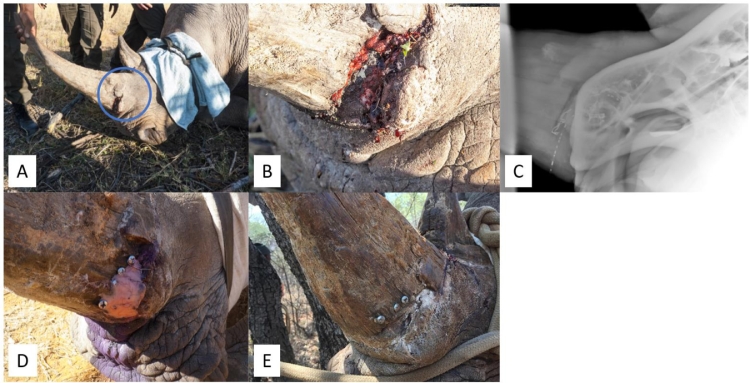
Injury of unknown origin at the base of the front horn, with extent to paranasal sinuses and signs of infection. A). Adult white rhino cow with a wound on the left side of the base of the front horn, see blue circle. B). Close-up picture of the wound at the start of treatment. C). Evaluation of the wound using contrast fluid and X-ray. D). Treatment of the wound with MGH and covering with dental acrylic polymer and screws. E). Fully healed result after 4 weeks, no signs of nasal discharge.

**Case 4:** An adult White Rhinoceros cow presented with an injury of unknown origin at the base of her front horn (Fig. 4A and 1B). The wound was suspected to be infected because of the presence of left nasal discharge, and this was confirmed by X-ray with contrast medium (Fig. 4C). On the X-ray, it is visible that the contrast medium entered the wound and extended to the bottom of the back horn. The wound was debrided, flushed and treated with MGH. The wound was subsequently covered with dental acrylic polymer and screws and wire, as standard bandaging and suture material is impossible to use in such location (Fig. 4D). Antibiotic therapy was not considered necessary and only anti-inflammatory drugs were administered. After 4 weeks the cow was immobilized, and the dental acrylic was removed. At this timepoint, there were no signs of a nasal discharge, and the wound healed exceptionally well, especially in comparison to what can be expected without using MGH (Fig. 4E). The screws and wire were removed, and the cow was woken up and released into the wild.

**Fig. 5:**
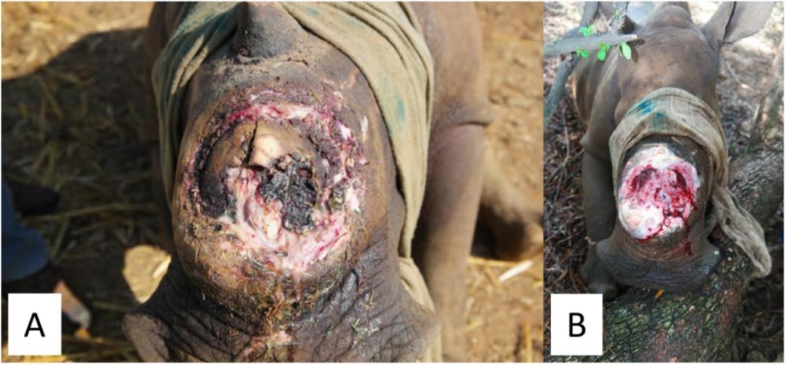
**A broken horn and fractures of the underlying nasal bone, accompanied with signs of infection. A).** The wound with visible areas of slough and devitalized tissue at the start of MGH therapy. **B).** Wound progression after 6 weeks of MGH treatment, with necrotic tissue replaced by healthy granulation tissue.

**Case 5:** A sub-adult White Rhinoceros heifer presented with a broken horn, accompanied by fractures of the underlying nasal bone. The wound was visibly infected, with areas of necrotic tissue and slough (Fig. 5A). The area was cleansed and MGH gel was applied and covered with fiberglass. After six weeks, all necrotic tissue was autolytic debrided and there was healthy granulation tissue present (Fig. 5B).

**Fig. 6:**
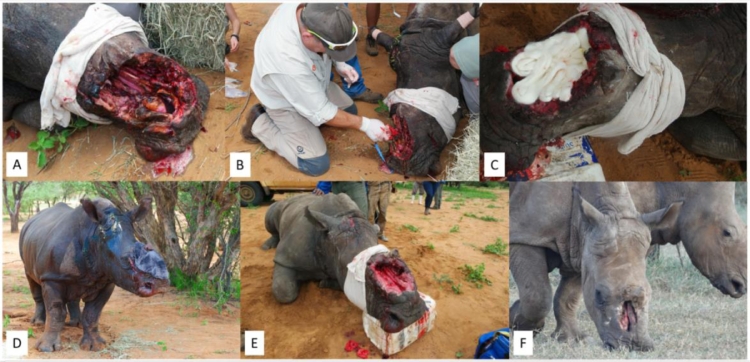
Gunshot wound through the head and severe damage and loss of tissue following hacking off the horns. A). Wound upon initial presentation. B). Cleaning and treatment of the wound with MGH. C). Application of alginate dressing to cover MGH. D). Elephant skin was placed over the wound to cover it. E). Clear wound progression with abundant areas of granulation tissue following the treatment with MGH. F). Complete healing of the wound after only 6 months.

**Case 6:** A two-year-old White Rhinoceros bull was shot through his head with another young female. The female died but fortunately he survived, but both front horns were hacked off, resulting in severe tissue damage and loss (Fig. 6A). The wound was cleaned and treated with MGH (Fig. 6B), dressed with calcium alginate (Fig. 6C) and elephant skin was sutured on top for protection and keeping the wound covered (Fig. 6D). This procedure was repeated several times. On follow up three months later, the wound had improved significantly with absence of infection and large areas of healthy granulation tissue (Fig. 6E). The wound healed extremely well within a total of 6 months (Fig. 6F).

**Fig. 7:**
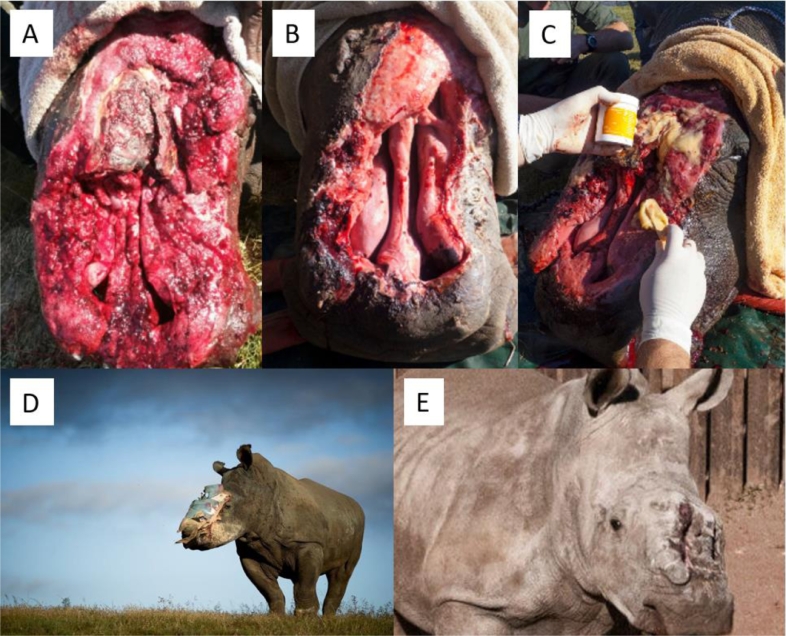
**The Rhino “Hope” with a severe and infected wound covering a large part of her head. A).** Start of the wound treatment with MGH **B).** The wound after four months of treatment with MGH. **C).** Example of MGH application. **D).** There is “Hope” for severely wounded rhinos. **E).** Hope almost fully recovered from the wounds and died 18 months after the poaching event due to an unrelated intestinal infection.

**Case 7:** A 5‐year-old White Rhinoceros cow who was shot and mutilated by poachers to have her front and back horns removed and left for death. There was a gigantic wound exposing the sinus cavities and nasal passage and presence of a massive infection in her facial area (Fig. 7A). Because of the severity of the injury, she was named “Hope”. The wound was repeatedly treated with MGH in combination with a calcium alginate dressing, resulting in remarkable healing within four months (Fig. 7B). An example of the application of MGH to the wound can be seen in Fig. 7C. There is still “Hope” for the most severely injured rhinos when these animals are treated with MGH (Fig. 7D). Hope survived the vicious attack and with intense veterinary care made it through three vital operations and almost fully recovered from the wounds (Fig. 7E). Unfortunately, she died of an unrelated bacterial infection of her small intestine after having lived 18 months after her first surgery.

## 3 Discussion

The White rhinoceros (*Ceratotherum simum*), along with other large African mega-herbivores, such as the African elephant (*Loxodonta africana*) are notorious victims of human persecution for its highly valuable tusks and horns (Duffy, St John, Buscher & Brockington, 2016; Ferreira et al., 2012). This tragic scenario was not always so, such that after a period of extinction and subsequent reintroduction into Kruger National Park in the 1960′s, rhino population numbers steadily increased (Ferreira et al., 2015). However, the last decade, poaching and the illegal activities on horns has found its way, resulting in a decrease of rhinos year over year. Unfortunately, poaching and natural mortality rates due to drought outpaced the birth of new rhinos (Ferreira et al., 2019). Maximal efforts need to be taken to turn the tide and prevention of poaching is key, and otherwise everything needs to be done to help the victims of poaching to survive, including optimizing wound care. An optimal wound care treatment would a simple and effective, also when provided irregularly. In this case series, we described the difficulties of treating the injured rhinos in their natural habitats in a frequent and regular manner and demonstrated the efficacy of MGH.

Since the establishment of “Saving the Survivors” in 2012, approximately 50 rhino per year have roughly been helped and released back in the wild. This, together with decades of experience of the different veterinarians (of which some more than 25 years on its own) has brought a very good knowledge on the wound healing process in rhinos. Although, each wound is unique, their experience provides a good insight on the expected healing trajectory, and they are unanimous that MGH will improve wound healing in rhinos. There is extensive literature that demonstrate the multiple mechanisms of MGH in wound healing, and its efficacy is supported in randomized controlled trials (Gustafsson et al., 2020; Mandel, Sutton, Abu & Kelmer, 2020). In a recent review paper about honey in wound care, 30 publications were discussed and it was concluded that honey shortened the time of wound healing and was cost-effective.(Yilmaz & Aygin, 2020)

Recently, Mandel et al. investigated whether a single intralesional application of MGH (L-Mesitran) would reduce the incidence of infection and dehiscence following surgically treated lacerations in horses (Mandel et al., 2020). This was evaluated in a prospective, open-label randomized clinical study in 127 horses (58 control cases and 69 treated with l-Mesitran) and infection rates were significantly decreased (OR: 3.64, CI: 1.42–9.26, *p* = 0.007) and wound healing was significantly improved (OR: 3.4, CI: 1.41–8.2, *p* = 0.006), when compared to control cases. Thus, a single subcutaneous application of MGH below the sutures of primary closed lacerations may be beneficial in preventing infection and dehiscence (Mandel et al., 2020).

In a subsequent study, Gustafsson et al. investigated whether subcutaneous application of MGH before the primary closure of colic surgeries in horses was safe and effective against surgical site infections (Gustafsson et al., 2020). In this clinical prospective randomized controlled trial, 108 horses were randomized to either control (no treatment) or treatment (a single subcutaneous application of MGH) group, after which 89 horses passed the inclusion and exclusion criteria, resulting in 49 horses in the treatment group and 40 horses in the control group. In total, 17 from the 89 horses were infected, of which 14 (32.5%) in the control group and 4 (8.1%) in the treatment group. The difference between was significant (*p* = 0.02) and MGH has a protective factor of 0.256 (CI: 0.084–0.834). Thus, horses that received MGH were four times less likely to suffer from a surgical site infection. No adverse reactions were observed with the subcutaneous application of MGH(Gustafsson et al., 2020).

Based on the presented results, other studies and our experience, we are confident that MGH has improved the wound healing in rhinos. Although it is not possible to treat these rhinos regularly, a single application has shown to resolve an infection without the use of antibiotics (case 2), which is in line with the equine studies described above. Also, in the other presented cases with (signs of) an infection (case 4, 5, 6, and 7), the infection was completely resolved. In humans, diabetic foot ulcers and diverse non-healing wounds infected with (multi-resistant) bacteria were effectively treated with MGH, while other therapies, including antibiotics, were ineffective (Holubová, Chlupácová, Cetlová, Cremers & Pokorná, 2021; Nair et al., 2020). Moreover, persistent biofilms were effectively treated with l-Mesitran Soft in the clinic (Pleeging et al., 2020).

A 100-fold difference in antimicrobial activity between honey types is reported (Mandal & Mandal, 2011). Although manuka honey may be extensively investigated, other types of honey have similar or stronger antimicrobial activity and may be more effective for wound healing (Cremers et al., 2020; Grego et al., 2016; Lusby, Coombes & Wilkinson, 2005; Majtan, Bohova, Prochazka & Klaudiny, 2013; Pleeging et al., 2020; Sherlock et al., 2010; Smaropoulos & Cremers, 2021). In a direct in vitro comparison study, l-Mesitran Soft had a stronger antimicrobial activity than manuka honey-based Medihoney against Staphylococci and Pseudomonas spp. pathogens, despite containing half the concentration of honey (Nair et al., 2020). This may be attributed to the different type of honey used or by the supplements that are added to the l-Mesitran Soft formulation. This enhanced antimicrobial activity has been observed in other studies comparing the l-Mesitran Soft formulation with only its raw honey (de Groot et al., 2021; Hermanns et al., 2019; Oliveira, Devesa & Hill, 2018). Recently, a synergistic activity of the supplements in l-Mesitran Soft has been confirmed in an in vitro biofilm wound model where l-Mesitran Soft was able to inhibit and eradicate *Pseudomonas aeruginosa* and *Staphylococcus aureus* bacteria biofilms (Pleeging et al., 2020).

The beneficial activity of MGH in wound care is based on two main pillars: its antimicrobial activity and wound healing activity. Both are subsequently based on multiple facets. The wide variety of mechanisms make MGH a versatile wound care product that can be used for all phases of wound healing. This has been clearly demonstrated by the presented cases. MGH can be used for treating locally infected wounds, including those with biofilms. MGH stimulates autolytic debridement of biofilms and necrotic and non-vital sloughy tissue, e.g. as clearly demonstrated in case 5. MGH stimulates autolytic debridement by creating a moist wound environment, acidification of the wound bed, and its osmotic activity that provides a constant supply of proteases (such as plasmin) at the interface of the wound bed and overlying necrotic tissue (Molan & Rhodes, 2015; Smaropoulos & Cremers, 2021). MGH has anti-inflammatory activity, and promotes healing by stimulating angiogenesis, granulation tissue formation, and re-epithelialization (E. Smaropoulos & N. A. Cremers, 2020; E. Smaropoulos & N. A. J. Cremers, 2020; Nair et al., 2020; Smaropoulos & Cremers, 2021). Topical application of MGH provides a rich nutrient source for proliferating cells that are rebuilding the lost tissue and promotes migration of new skin cells (E. Smaropoulos & N. A. Cremers, 2020). Randomized controlled trials to substantiate these findings are hard to perform due to ethical considerations, the scarcity of cases, the difficulties with follow-up, and each wound being unique. These difficulties also explain why a case series of a limited sample size is presented to illustrate the potency of MGH, rather than performing a large case-control study which would be not compatible with the subjects.

We are confident that this case series provide a valuable insight into the wound healing process of these majestic animals. Having such knowledge is of importance when these patients present with difficult and often life-threatening injuries.

## 4 Conclusion

The number of rhinos in the wild is decreasing year over year by poaching and drought. Poaching needs to be prevented and otherwise minimized with all possible efforts. When rhinos are poached and left for death, it is important to save the survivors with an easy and effective wound care product. MGH holds both antimicrobial and healing activities and forms a potent and promising treatment. As supported by the presented cases, MGH effectively improves wound healing via multiple mechanisms and provides hope to even the most severely injured rhinos.

## Data accessibility statement

All data is provided in full in the results section of this paper. No other publicly accessible repository is used for storage of the data.

## Ethical statement

This work involved the treatment of wounded animals in the wild, and thus non-experimental animals only. The MGH used in this study is a registered wound care product (CE and FDA approved). Since the treatment is part of regular care and follows the instructions for use of the product, no ethical approval was necessary to treat these endangered wounded wild animals.

## Declaration of Competing Interest

NC is employed by Triticum Exploitatie BV. Triticum Exploitatie BV is the manufacturer of the medical grade honey formulation l-Mesitran. Triticum Exploitatie BV provided the l-Mesitran used in the study free of charge and sponsors the non-profit organization “Saving the Survivors”. “Saving the Survivors” treats endangered wild animals that have fallen victim to poaching or traumatic incidents. NC was not involved in the design of the study, the treatment of the animals or presentation of the results. No other conflict of interest applies.
